# Cultivable Bacterial Communities in Brines from Perennially Ice-Covered and Pristine Antarctic Lakes: Ecological and Biotechnological Implications

**DOI:** 10.3390/microorganisms8060819

**Published:** 2020-05-29

**Authors:** Carmen Rizzo, Antonella Conte, Maurizio Azzaro, Maria Papale, Alessandro C. Rappazzo, Dario Battistel, Marco Roman, Angelina Lo Giudice, Mauro Guglielmin

**Affiliations:** 1 Stazione Zoologica Anton Dohrn, Department of Marine Biotechnology, National Institute of Biology, Villa Pace, 98167 Messina, Italy; carmen.rizzo@szn.it; 2 Department of Chemical, Biological, Pharmaceutical and Environmental Sciences, University of Messina, 98166 Messina, Italy; conte.antonella@outlook.com; 3 Institute of Polar Sciences, National Research Council (ISP-CNR), 98122 Messina, Italy; maurizio.azzaro@cnr.it (M.A.); mpapale@unime.it (M.P.); rappale@libero.it (A.C.R.); 4 Dipartimento di Scienze Ambientali, Informatica e Statistica, University Ca’ Foscari, 30123 Venezia, Italy; dario.battistel@unive.it (D.B.); marco.roman@unive.it (M.R.); 5 Dipartimento di Scienze Teoriche e Applicate, University of Insubria, 21100 Varese, Italy; mauro.guglielmin@uninsubria.it

**Keywords:** brine lenses, Antarctic cultivable bacteria, contaminants, bioprospecting

## Abstract

The diversity and biotechnological potentialities of bacterial isolates from brines of three Antarctic lakes of the Northern Victoria Land (namely Boulder Clay and Tarn Flat areas) were first explored. Cultivable bacterial communities were analysed mainly in terms of bacterial response to contaminants (i.e., antibiotics and heavy metals) and oxidation of contaminants (i.e., aliphatic and aromatic hydrocarbons and polychlorobiphenyls). Moreover, the biosynthesis of biomolecules (antibiotics, extracellular polymeric substances and enzymes) with applications for human health and environmental protection was assayed. A total of 74 and 141 isolates were retrieved from Boulder Clay and Tarn Flat brines, respectively. Based on 16S rRNA gene sequence similarities, bacterial isolates represented three phyla, namely Proteobacteria (i.e., Gamma- and Alphaproteobacteria), Bacteroidetes and Actinobacteria, with differences encountered among brines. At genus level, *Rhodobacter*, *Pseudomonas*, *Psychrobacter* and *Leifsonia* members were dominant. Results obtained from this study on the physiological and enzymatic features of cold-adapted isolates from Antarctic lake brines provide interesting prospects for possible applications in the biotechnological field through future targeted surveys. Finally, findings on contaminant occurrence and bacterial response suggest that bacteria might be used as bioindicators for tracking human footprints in these remote polar areas.

## 1. Introduction

Brine lenses are small amounts of water that remains unfrozen below 0 °C due to its high salt content. In Antarctica, brines are possibly concealed in a number of cryosystems, such as glaciers, subglacial and ice-covered lakes and permafrost [1,2,3,4,5,6,7]. By a geophysical point of view, Antarctic subglacial streams connect lakes and ponds with their movements, by making brine systems highly dynamic [5,8,9]. However, mobilization processes, as well as the brine genesis, remain poorly understood [10]. Interestingly, the similarities of brine pockets with conditions retrieved on Mars, in terms of cold and dry climates, together with the development of highly saline microhabitat [11,12], lead researchers to question the common meaning of limits of life and brines are suggested as a model to study extra-terrestrial life [6,13].

Beside their high geological and astrobiological relevance, briny ecosystems are oases for extant microbiota, which is able to withstand the high salt concentrations and osmotic pressure (e.g., protection from cell desiccation) and low temperature (e.g., by the production of cryoprotective agents) [1]. The extreme multifaceted environmental constrains of Antarctic brines make them ideal habitats for the exploration of microbial biodiversity and evolution, through a deep comprehension of adaptive and survival strategies adopted by inhabiting microorganisms, including the biosynthesis of a plethora of enzymes or other bioproducts of biotechnological interest. Being pristine, extreme and almost undisturbed environments, Antarctic lake brine pockets may also represent key habitats for Antarctic microbial diversity monitoring in relation to climate change and the unavoidable incoming contamination. Despite its remoteness and the measures adopted to precautionary avoid its contamination, the Antarctic continent can be reached by a plethora anthropogenic pollutants through long-range transport by mass flow in the atmosphere and water (at global scale) and/or human activities at research bases [14,15], thus making pollution a worrying issue [16,17]. Heavy metals, hydrocarbons and PCBs are mong contaminants commonly reported in Antarctic matrices and, as it is well known, microorganisms can adapt and respond to the growing presence of toxic compounds in the environment [15,18,19,20].

**Figure 1:**
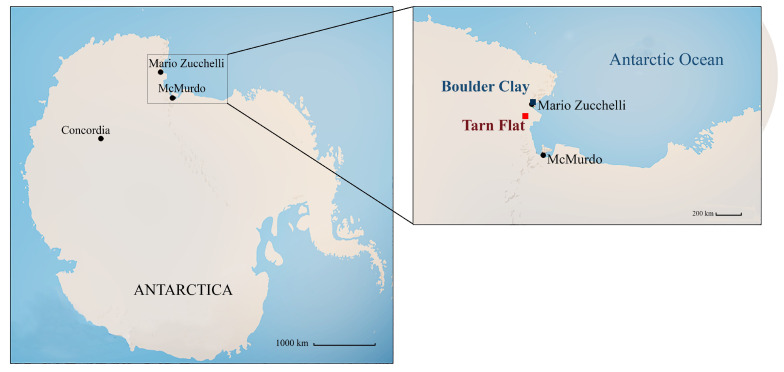
Tarn Flat and Boulder Clay sites in Antarctica.

Brine lenses were detected within the perennial ice-covers of Antarctic lakes lying in the Northern Victoria Land, namely in the Boulder Clay [21,22,23] and Tarn Flat [6] areas (Figure 1).

Tarn Flat is an ice-free area (ca. 100 km^2^) in the north to the McMurdo Dry Valley in Victoria Land characterized by the presence of many lakes, at different degrees of freezing dependent on the altitude and the season [6,24]. The incidence of katabatic winds, the low precipitation rate [25] and the presence of glaciers contribute to the occurrence of a cold and arid climate, with a mean monthly temperature that ranges between 2−6 and 0 °C and a mean annual temperature of 1−3.9 °C. Boulder Clay is an ice-free area located in a slope with south-eastern exposure, characterized by perennially ice-covered ponds with icing blisters and frost mounds [23,26], frost-fissure polygons and debris island [22]. The soils here are mainly glacic haplorthels where no evidence of cryoturbation has been observed and scattered mosses and epilithic lichens constitute the vegetation [27]. The area is affected by katabatic winds, with mean annual temperature at 1−3.8 °C. A lot of geological formations of different nature and dating back to the Pleistocene have been documented in the area [28]. Recently, the Boulder Clay moraine was chosen as location for building a new semi-permanent gravel runway for airfreight operations by the Italian National Antarctic Research Program.

Recently, brine lenses from Tarn Flat and Boulder Clay lakes were explored for prokaryotic and eukaryotic diversity and activities by the application of advanced culture-independent methods [13,29,30,31]. Exceptionally diverse (in terms of both ecophysiology and phylogenetic affiliation) prokaryotic communities, which included methanogens, strictly anaerobes, halophiles and (hyper)thermophiles, were characterized [13,31]. As a complementary research, bacterial isolates were also obtained from those brine samples. The main aim of the present study was thus the exploration of ecological and biotechnological features of the cultivable fraction of the bacterial communities in brine lenses of three Antarctic lakes, two of which located in the Boulder Clay and one in the Tarn Flat areas, respectively. We mainly report on (i) the abundance and diversity of bacterial communities recovered from brine lenses, (ii) their tolerance to heavy metals and susceptibility to antibiotics, (iii) their ability to growth in presence of organic contaminants (i.e., aliphatic and aromatic hydrocarbons and polychlorinated biphenyls) and (iv) their bioprospective potential with regard to cold-active enzymes, antibiotics and extracellular polymeric substances.

## 2. Materials and Methods 

### 2.1. Study Areas and Sample Collection

Brines were collected from three perennially frozen Antarctic lakes, lying in two sites of the Northern Victoria Land, namely Tarn Flat (TF; coordinates: 75°4′ S 162°30′ E) and Boulder Clay (BC; coordinates: 74°44′ S–164°01′ E). Brine samples were collected near the ice blisters of the adjacent Lake 16 (L16; i.e., brines BC1 and BC2 from two different sampling points at a depth of 2.5 and 1.0 m, respectively) and Lake L-2 (i.e., brine BC3, depth 2.0 m) in the Boulder Clay area [30,31] and from a perennially frozen lake at the highest elevation of the Tarn Flat area, namely brines TF4 (between 3.78 and 3.98 m depth) and TF5 (between 4.10 and 4.94 m depth) along the same borehole [6,13]. All samples were collected in pre-sterilized polycarbonate bottles by using sterilized peristaltic pump and tubing. The main physico-chemical features of TF and BC brines are reported in Forte et al. [6] and Azzaro et al. [30], respectively.

### 2.2. Chemical Analyses

Trace elements were determined in the brine liquid fractions. Brine aliquots (50 mL) were immediately mixed and frozen in polyethylene tubes for storage until analysis. Upon unfreezing, samples were shortly vortex-mixed and then centrifuged at 3000 rcf for 5 min to settle the suspended particles. The liquid fraction was transferred into new tubes, acidified and possibly diluted (to decrease the saline content) as follows—samples BC2 and BC3 were spiked with concentrated ultrapure grade HNO_3_ to 2% (*v/v*) final concentration of the acid; samples BC1, TF4 and TF5 were diluted 1:10 (*v/v*) with Milli-Q water (18 MΩ cm^−1^ at 25 °C, TOC ≤1 ppb) and ultrapure grade HNO_3_ to 2% (*v/v*) final concentration of the acid. The solutions were directly analysed for determination of cobalt, nickel, copper, cadmium and mercury. The multielemental analysis was carried out by inductively coupled plasma-mass spectrometry (ICP-MS) using an instrument Thermo Scientific (Venezia, Italy) iCAP RQ equipped with ASX-560 autosampler (Teledyne Cetac Technologies, Omaha, NE, USA), glass cyclonic spray chamber thermostated at 2.7 °C, quartz torch, nickel cones and 1550 W of plasma RF power. The acquisition was performed in Kinetic Energy Discrimination—high sensitivity (KEDS) mode using He as the collision gas (4.3 mL min^−1^). Instrumental parameters were optimized for best sensitivity in the whole mass range and minimum oxides (<1%) and double charges (<3%) levels. The monitored *m/z* were 59 (Co), 60 (Ni), 63 (Cu), 111 (Cd) and 202 (Hg). Quantification was obtained by external calibration with multielemental standards prepared in ultrapure grade HNO_3_ 2% *v/v* and Milli-Q water, from the certified level multielemental solution IMS-102 (UltraScientific, Bologna, Italy). On-line spiked Rh (*m/z* 113) was used as the internal standard to compensate for possible matrix effects and instrumental drifts during the analysis. The internal standard spike solution was prepared in ultrapure grade HNO_3_ 2% *v/v* and Milli-Q water from a certified level Rh solution (UltraScientific, Bologna, Italy). Three repeated acquisitions were performed for all elements within each analysis.

The same aliquots previously analysed for trace elements were also considered for organic pollutants such as volatile *n*-alkanes and polycyclic aromatic hydrocarbons (PAHs) through direct immersion solid-phase microextraction (DI-SMPE) adapting the method reported in Lorenzo-Parodi et al. [32] and references therein. The SPME was performed using 50/30 µm divinylbenzene/carboxen/polydimethylsiloxane (DVI/CAR/PDMS) fibres (Supelco, Bellefonte, Bellefonte, PA, USA) and an extraction time of 30 min. The organic pollutants were determined by gas chromatography-mass spectrometry (GC-MS) with a 6890-N-GC system coupled to a qMS 5973 detector (Agilent Technologies, Santa Clara, CA, USA). A series of 3 procedural blanks was carried out in 50 mL of Milli-Q water. Procedural limits of quantification (LOQ) were ~100 ng L^−1^ and ~80 ng L^−1^ for *n*-alkanes and PAHs, respectively.

### 2.3. Cultivable Bacterial Community 

#### 2.3.1. Isolation of Heterotrophic Bacteria

Aliquots (200 μL) of each brine sample were spread-plated in duplicate on different solidified culture media, as follows—Tryptic Soy Agar at full (TSA_100_; Oxoid, Italy), 1/2 (TSA_50_) and 1/10 (TSA_1_) strength; R2A agar (Difco, Milano, Italy) at 1/5 (R2A_20_) and 1/10 (R2A_10_) strength; AcidAgar (composition *per* litre—NaCl, 0.813 g; glucose, 1.0 g; K_2_HPO_4_ × 3H_2_O, 0.86 g; MgSO_4_ × 7H_2_O, 0.5 g; yeast extract, 1.0 g; agar, 15.0 g; pH 4); DSMZ 97 (composition *per* liter—NaCl, 250.0 g; MgSO_4_ × 7H_2_O, 20.0 g; KCl, 2.0 g; sodium citrate, 3.0 g; casaminic acid, 7.5 g; yeast extract, 1.0 g; FeSO_4_, 0.00023 g; agar, 15.0 g; polimixyn B, 50 mg mL^−1^, 0.75 g; pH 7.35) and DSMZ 371 (composition *per* liter—KH_2_PO_4_, 1.0 g; KCl, 1.0 g; NH_4_Cl, 1.0 g; MgSO_4_ × 7H_2_O, 0.24 g; CaSO_4_ × 2H_2_O, 0.17 g; SL1−0 solution; NaCl, 200.0 g; Na_2_-glutammate, 1.0 g; yeast extract, 5.0 g; casaminic acid, 5.0 g; Na_2_CO_3_, 5.0 g; pH 6.5). Agar plates were incubated in the dark at 4 °C for 8 weeks. Colony forming units per mL of brine samples (CFUs mL^−1^) were calculated as averages of duplicate plates. For bacterial isolation, colonies were randomly selected from agar plates used for CFU counts, picked and subcultured almost three times under the same conditions.

#### 2.3.2. PCR-Amplification of 16S rRNA Genes

PCR-amplification of 16S rRNA genes from bacterial isolates was carried out using the domain Bacteria-specific primers 27F (5′-AGAGTTTGATCCTGGCTCAG-3′) (spanning positions from 8 to 27 in *E. coli* rRNA coordinates) and 1492R (5′-CTACGGCTACCTTGTTACGA-3′) (spanning positions from 1492 to 1513 in *E. coli*) under the conditions described earlier [33]. 

#### 2.3.3. Sequencing and Analysis of the 16S rRNA Genes

Sequencing was carried out at the Sequencing Service of the Macrogen Laboratory (Amsterdam, The Netherlands). The closest relatives of isolates were determined by comparison to 16S rRNA gene sequences in the NCBI GenBank and the EMBL databases using BLAST [34] and the “Seqmatch” and “Classifier” programs of the Ribosomal Database Project II (http://rdp.cme.msu.edu/). The program Clustal W was used to further align sequences with the most similar orthologous sequences [35] detected from database. According to the Jukes-Cantor distances model, each alignment was verified and corrected using the Neighbour-Joining method [36]. All sequences with similarity ≥97% were considered to represent one phylotype and were grouped into Operational Taxonomic Units (OTUs). Isolates belong to the Italian Collection of Antarctic Bacteria (CIBAN) of the National Antarctic Museum (MNA). 

#### 2.3.4. Culture Conditions for Bacterial Growth

Cultures were incubated at 4 °C for 21 days, unless differently specified. Bacterial growth was assayed at different temperature (4, 15 and 25 °C) and pH (4, 5, 6, 7, 8 and 9) values in Nutrient Broth (NB; Oxoid). The pH of the medium was adjusted by the addition of HCl and NaOH (0.01 g L^−1^, 0.1 g L^−1^ and 1 g L^−1^ solutions). Salt tolerance tests were performed on Nutrient Agar (NA; Oxoid) with NaCl concentration ranging from 0 to 19% (*w/v*). 

### 2.4. Antibiotic Susceptibility and Heavy Metal Tolerance

#### 2.4.1. Antibiotic Susceptibility

Antibiotic susceptibility was determined on TSA plates supplemented with antibiotics at different concentrations, as follows—ampicillin (AMP; 50 and 100 ppm), kanamycin (KAN; 50 and 100 ppm), streptomycin (STR; 25, 250 and 350 ppm), chloramphenicol (CHL; 50 and 100 ppm), gentamycin (GEN; 25, 250 and 350 ppm) and polymyxin B (PB; 50 and 100 ppm). These concentrations were chosen on the basis of previous results on antibiotic tolerance by Antarctic bacteria [37,38,39]. A stock solution of each antibiotic was prepared and added to sterile TSA at 50 °C to avoid damages of the molecules. Plates were inoculated by streaking and incubated at 4 °C for 21 days.

#### 2.4.2. Heavy Metal Tolerance

Heavy metals (HMs) tolerance was verified by seeding bacterial strains on TSA supplemented with heavy metal salts (i.e., CdCl_2_, CuCl_2_, HgCl_2_, CoCl_2_ and NiCl_2_) at both 500 and 1000 ppm. A stock solution at 10,000 or 20,000 ppm in 1× phosphate-buffered saline (PBS) was prepared for each heavy metal salt and added before sterilization to the medium to final concentration of 500 or 1000 ppm [38,39,40,41]. Isolates were streaked on TSA plates plus HMs and incubated at 4 °C for 21 days. Strains that were able to grow until 1000 ppm of HMs were streaked on TSA medium amended with the same metal until a concentration of 10,000 ppm.

### 2.5. Biotechnological Potential of Bacterial Isolates

For the following assays, cultures were incubated at 4 °C for 21 days, unless otherwise specified. 

#### 2.5.1. Oxidation of Hydrocarbons

The bacterial ability to grow in the presence of hydrocarbons as the only carbon source and energy was tested for solid (i.e., naphthalene, phenanthrene, pyrene) and liquid hydrocarbons (i.e., toluene, tetradecane, heptane, octane, dodecane), both aliphatic and aromatic substrates, as well as for two hydrocarbon mixtures, that is, crude oil and diesel oil [42,43,44]. Strains were streaked on the mineral medium Bushnell Haas (BH; DIFCO, Milano, Italy) added with 3% NaCl (*w/v*) and 1.5% agar (*w/v*) and hydrocarbons were placed on the lid of the Petri dish. In the case of liquid hydrocarbons and hydrocarbon mixtures, a cellulose pad was soaked with a 100 μL-aliquot of the substrate. 

Moreover, strains able to grow on plate in the presence of diesel oil and crude oil were further tested in liquid cultures. Sterile crude oil or diesel oil (final concentration 2.0%, *v/v*) as the sole carbon and energy source were added to liquid BH supplemented with 3% NaCl (*w/v*). The medium was inoculated with 10% of a freshly prepared bacterial suspension in 3% (*w/v*) NaCl-supplemented BH medium. After incubation, the ability to use hydrocarbons as growth substrates was evaluated according to the degree of turbidity or the appearance of cellular flocs in the test tubes. Uninoculated medium was incubated in parallel as a negative control.

#### 2.5.2. Oxidation of Polychlorobiphenyls

Bacterial isolates were screened for growth on Aroclor 1242 (Sigma-Aldrich, St. Louis, MO, USA), a mixture of PCB congeners (ranging from dichloro- to hexachlorobiphenyls) made of twelve carbon atoms in the biphenyl molecule and containing 42% chlorine by weight [45]. Aroclor 1242 (100 ppm in dichloromethane) was added as sole carbon and energy source (final concentration 0.1%, *wt/vol*) to liquid BH and isolates tested for growth under the conditions reported by Michaud et al. [46]. After incubation, the ability to use PCBs as growth substrates was evaluated according to the degree of turbidity or the appearance of cellular flocs in the test tubes. Uninoculated medium was incubated in parallel as a negative control. Finally, isolates growing in presence of Aroclor 1242 were further screened for the presence of the catabolic gene *bph*A involved in PCB degradation, under the conditions reported by Papale et al. [47]. 

#### 2.5.3. Extracellular Enzymes and Hydrolysis of Complex Substrata

For DNA hydrolysis capacity, strains were streaked on DNase agar (Oxoid). The presence of DNAse was confirmed by the appearance of clarification halo around the colonies after using hydrochloric acid 1 N as indicator. *Staphylococcus aureus* ATCC23235 was used as a positive control. The hydrolysis of other complex substrata (i.e., chitin, agar, tween 80, gelatine and starch) was assayed according to previously reported methods [48,49].

#### 2.5.4. Inhibition of Bacterial Growth

Brine isolates were screened for their antagonistic activity towards a selection of target microorganisms, as follows—*Escherichia coli* ATCC25922, *Pseudomonas aeruginosa* ATCC27853, *Staphylococcus aureus* ATCC23235, *Micrococcus luteus* ATCC4698, *Bacillus subtilis* ATCC6051, *Salmonella enterica* ATCC35664. Experiment was performed on TSA at 1.5% of NaCl. Antibacterial activity was detected by the cross-streak method as previously described [50,51]. Antarctic bacteria were streaked across one-third of an agar plate and incubated at 15 °C. After that growth of test strain occurred (generally within 710-days), indicator organisms were streaked perpendicularly to the initial streak and plates were further incubated at 37 °C for 72/120 h and checked afterwards for the appearance of inhibition zones. The antagonistic effect was indicated by the failure of the target strain to grow in the confluence area.

#### 2.5.5. Other Enzymes

To test haemolytic activity strains were streaked on Blood Agar medium (Oxoid), containing 5% of sheep blood and incubated. Cultures were incubated at 4 °C for 21 days. After incubation, plates were checked and positive results were indicated by the presence of halos. Catalase activity was detected by checking bubble production in a 3% (*v/v*) hydrogen peroxide solution, while oxidase activity was colorimetrically assayed using BBLTM DrySlideTM oxidase slides (DIFCO).

#### 2.5.6. Production of Extracellular Polymeric Substances

Bacterial isolates were streaked on TSA medium added with a glucose solution (3% *v/v*, final concentration) and incubated. After incubation, isolates showing a mucoid aspect were further subjected to the slime test in Tryptone Soy Broth (TSB; Oxoid) added with glucose (2%, *v/v*, final concentration) [52]. EPS production was quantified for selected isolates as reported by Caruso et al. [53] growing bacteria in Väätänen nine-salt solution (VNSS) amended with glucose (2%, *w/v*, final concentration). After incubation, the cultures were centrifuged at 8000 rpm for 10 min and EPS amounts in cell-free supernatants were evaluated by the phenol-sulphuric acid method [54].

### 2.6. Statistical Analyses

Data were analysed by one-way ANOVA to detect significant differences in the datasets from BC and TF brine samples (MiniTab software, State College, PA, USA; version 16.0; significance level 0.05). Bray Curtis similarity coefficients were computed on the entire dataset and used to perform Not-metric multi-dimensional scaling (nMDS) of the brines isolates. Principal component analysis (PCA) and cluster analysis were used to provide the visual grouping of the results from bacterial isolates in relation to the environmental parameters. All analyses were performed by using Primer 6 (Plymouth Marine Laboratory, Roborough, UK). Venn Diagram was constructed by using a web-based tool for the analysis of dataset of genera distribution [55].

## 3. Results

### 3.1. Chemical Analyses

**Figure 2:**
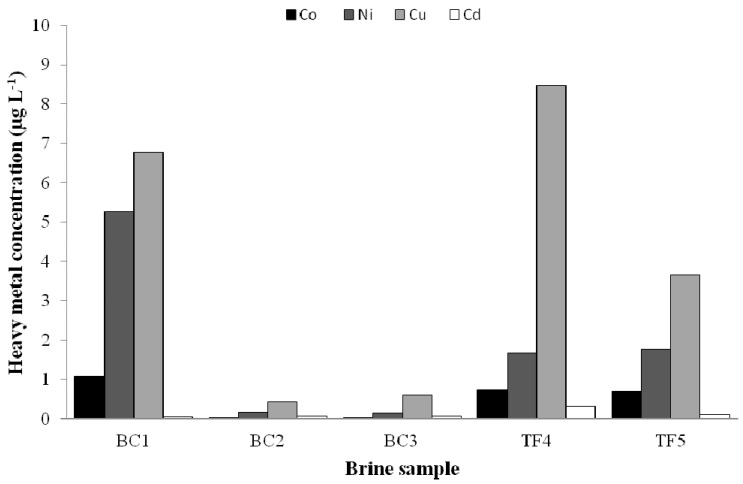
Trace metal concentrations in the Boulder Clay (BC) and Tarn Flat (TF) brine samples.

The concentrations of HMs detected in brine samples are reported in Figure 2. Overall, nickel and copper presented the higher levels, especially in TF (with a concentration of 1.7 and 1.8 µg L^−1^ for nickel and 8.5 and 3.7 µg L^−1^ for copper in TF4 and TF5 samples, respectively) and in BC1 brine samples (5.3 and 6.8 µg L^−1^ for nickel and copper, respectively). Cadmium was the less abundant element in all samples, while was detected with a concentration of 1 µg L^−1^ in BC1 brine samples. The concentration of mercury in all brines was lower than its procedural LOQ (50 ng L^−1^ for undiluted samples), compatibly to its expected low background level and possible losses of the element during storage/preparation due to high volatility. 

In all the samples analysed, the concentration of the more volatile aromatic and aliphatic hydrocarbons resulted below the LOQ previously reported in the Materials and Method section. The low concentrations of the more volatile organics may be ascribed to the fact that the brines represent a pristine environment and they have never been directly exposed to any pollution sources, although we cannot exclude a loss of the more volatile compounds during sampling operations or pre-analytical steps. In addition, the aqueous volume available may have been a limiting factor for the analysis in these particular samples. 

### 3.2. Cultivable Bacteria Community 

#### 3.2.1. Bacterial Isolation

*Boulder Clay*. As it is shown in Table 1, all BC samples yielded colonies on TSA media, with viable counts ranging between 0.0 and 15.5 × 10^3^ CFU mL^−1^ on TSA1 (BC3 and BC1, respectively), 0.5 and 4.3 × 10^3^ CFU mL^−1^ on TSA_50_ (BC2 and BC1, respectively) and 0.1 and 5.3 × 10^3^ CFU mL^−1^ on TSA_100_ (BC2 and BC1, respectively). BC3 showed growth on DSMZ 97 medium (0.1 × 10^3^ CFU mL^−1^) but no colonies were yielded. No growth was observed on R2A_20_ and DSMZ371 medium for BC brines. A total of 74 strains were isolated from BC brine samples (mainly from sample BC1 inoculated on the medium TSA1) (Table 1).

*Tarn Flat*. Both TF samples yielded colonies on TSA and R2A plates. No colonies were recovered from R2A_20_ and DSMZ agar medium. Viable counts on TSA media were 3.1 ± 0.8 and 7.2 ± 0.6 × 10^3^ CFU mL^−1^ (in TF4 and TF5, respectively) on TSA_1_, 4.7 ± 0.9 and 5.5 ± 0.0 × 10^3^ CFU mL^−1^ (in TF4 and TF5, respectively) on TSA_50_ and 3.3 ± 0.1 and 5.5 ± 0.8 × 10^3^ CFU mL^−1^ (in TF4 and TF5, respectively) on TSA_100_. Viable counts on R2A_10_ were 0.1 × 10^3^ CFU mL^−1^ in both samples. A total of 141 strains were isolated from TF brine samples, with 66 and 75 of them that were obtained from TF4 and TF5, respectively. In particular, 55 strains were recovered from TSA1 (14 and 41 from TF4 and TF5, respectively), 30 from TSA_50_ (16 and 14 from TF4 and TF5, respectively) and 47 from TSA_100_ (31 and 16 from TF4 and TF5, respectively). Finally, 9 strains were isolated from R2A_10_ (five and four from TF4 and TF5, respectively) (Table 1).

*Boulder Clay* vs. *Tarn Flat*. No statistical differences were detected between brines on viable counts on each culture medium. Not-metric multi-dimensional scaling (nMDS) performed on viable counts obtained from all brine samples highlights the higher isolation potential of rich media and the grouping of TF brine samples together with BC1 brine samples, the principal source of bacterial isolates in this work.

#### 3.2.2. Bacterial Identification

**Figure 3:**
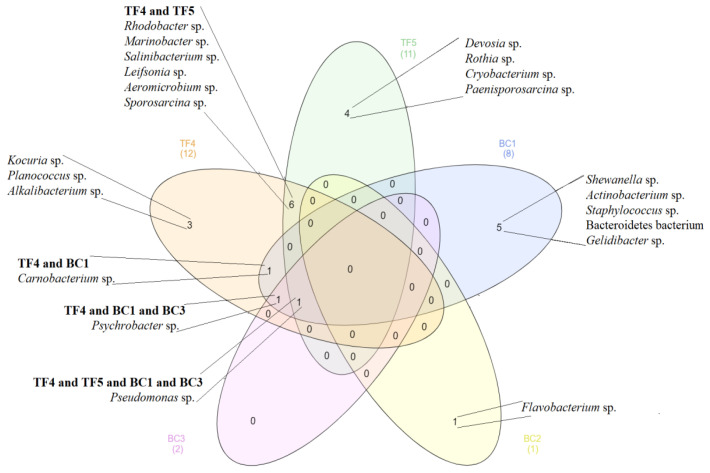
Venn Diagram constructed with data from the genera distribution of bacterial isolates among all brine samples.

The phylogenetic affiliation of the 215 bacterial isolates from Antarctic lake brines and their distribution among samples in terms of genera are reported in Table 2 and Figure 3. Overall, Proteobacteria members were dominant in all brine samples. They were exclusively represented by Gammaproteobacteria in BC brines, while Gamma- and Alphaproteobacteria were in both TF brines. Bacteroidetes members were retrieved only in BC brines, while a higher number of isolates affiliated to Actinobacteria were obtained from TF samples. A total of 27 phylotypes were detected in all brine samples. 

*Boulder Clay*. The 74 isolates from BC brines (mainly obtained from BC1; 64 isolates) grouped in nine OTUs, distributed across four bacterial phylogenetic groups, namely Gammaproteobacteria (70.3% of total isolates), Firmicutes (13.5%), CF group of Bacteroidetes (6.8%) and Actinobacteria (1.4%). Six sequences (four and two from BC1 and BC3, respectively; 8.2% of total isolates) were not affiliated. At genus level, *Pseudomonas* was predominant. OTU-sharing among BC brines was observed only between BC1 and BC3 samples for OTU24 and OTU27, affiliated to the genera *Pseudomonas* and *Psychrobacter*, respectively.

*Tarn Flat*. The 141 isolates from TF brines grouped in 18 separated phylotypes, which fell into well-defined phyla composing the bacterial lineage, as follows—Gammaproteobacteria (52.5% of total isolates), followed by Actinobacteria (20.6%), Alphaproteobacteria (15.6%) and Firmicutes (11.4%). The bacterial community composition differed between TF4 and TF5 samples, with the Gammaproteobacteria (71.2% vs. 36.0%) that strongly predominated over all other groups in TF4 and Alphaproteobacteria (3.0% vs. 26.7%) and Actinobacteria (12.1% vs. 28%) that occurred at higher percentages in TF5. Firmicutes were similarly represented in TF4 and TF5 (13.6% and 9.3%, respectively). OTU-sharing between TF brines was seldom observed (Table 2), with six OTUs that were common to both brine samples, that is, OTU1 (*Aeromicrobium* sp. TF4-24), OTU4 (*Leifsonia* sp. TF5-105B), OTU6 (*Marinobacter* sp. TF4-237), OTU9 (*Pseudomonas* sp. TF4-182), OTU13 (*Rhodobacter* sp. TF5-149) and OTU15 (*Sporosarcina* sp. TF4-168). Among them, members in the genera *Rhodobacter*, *Pseudomonas* and *Leifsonia* were more abundant in TF5 than in TF4. All remaining OTUs/phylotypes were obtained from individual samples (seven and five from TF4 and TF5, respectively). In particular, *Psychrobacter* isolates (32 isolates within OTU10 and *Psychrobacter* sp. TF4-164) were retrieved exclusively from TF4. 

Nucleotide sequences have been deposited in the GenBank database under the accession nos. KY437986-KY438003 (Tarn Flat isolates) and MT350301-MT350309 (Boulder Clay isolates).

#### 3.2.3. Growth Conditions of Bacterial Isolates

**Figure 4:**
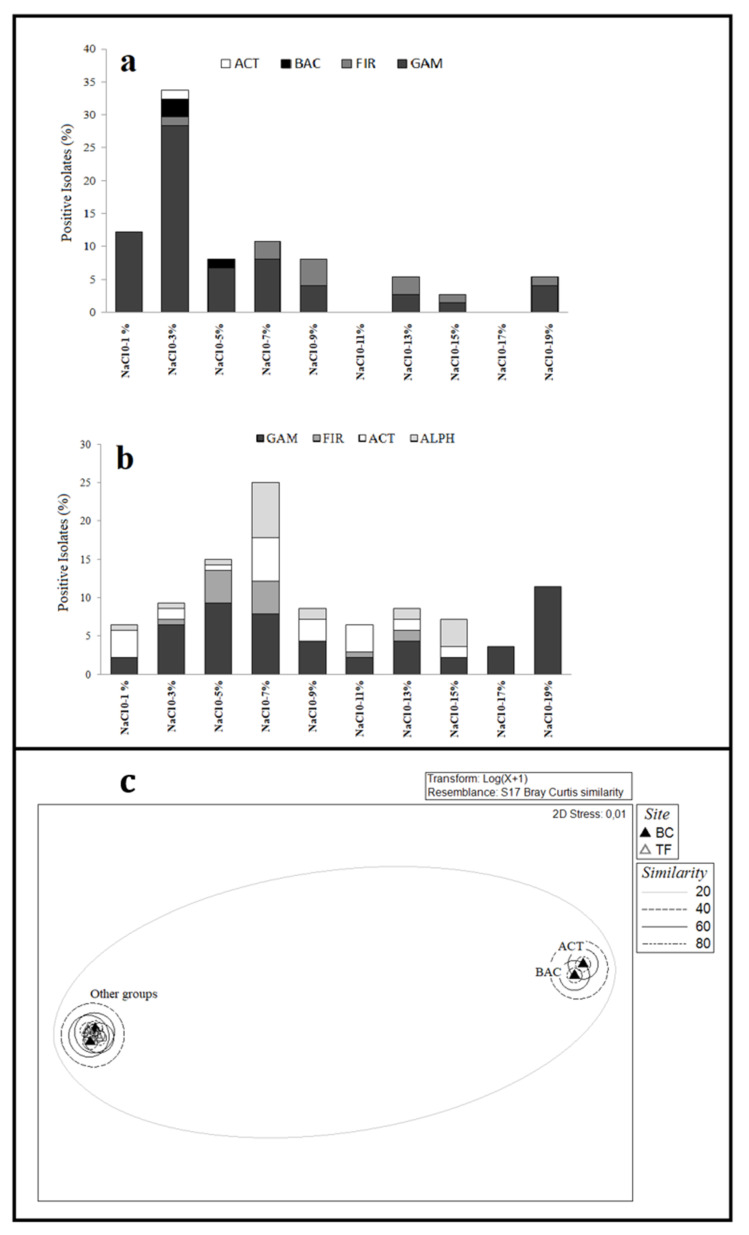
Bacterial growth at different salinity values for Boulder Clay brines (**a**) and Tarn Flat brines (**b**). Nonmetric multi-dimensional scaling analysis (nMDS) computed on transformed and clustered data by imposing the sampling site as a factor (**c**).

*Boulder Clay*. *Flavobacterium* isolates from BC2 did not survive to sub-culturing and they were not tested for optimal growth conditions. All isolates from BC brines were able to grow between 4 and 25 °C, with the exceptions of *Carnobacterium* sp. BC1-73 that grew only at 4 °C and three *Pseudomonas* spp. isolates which did not grow at 25 °C. The optimal pH range for most of the isolates was between 6/7 to 9. All isolates were able to grow in the absence of NaCl, with the majority of them that grew at salinity values up to 3% NaCl (*w/v*) (25 isolates; 33.8%). Four isolates, affiliated to the genera *Staphylococcus* and *Psychrobacter* (isolated from BC1 and BC3 brine samples) were able to grow up to 17–19% NaCl. In particular, isolates from BC1 brines better tolerated higher salinities with the 13.5% of strains (ten isolates) that grew up to 7% of NaCl (Figure 4a).

*Tarn Flat*. Isolates from TF brines, with few exceptions (six isolates growing only at 4 °C), were able to growth at both 4 and 25 °C. The optimal pH range for growth was from 6/7 to 9 for most isolates (80 isolates; 56.7%). All isolates were able to grow in the absence of NaCl, with the majority of them that grew in a wide salinity range and tolerated up to 15% (*w/v*) NaCl. A total of 22 isolates from TF4 (mainly in the genus *Psychrobacter*) were able to grow up to 17–19% NaCl (Figure 4b). 

*Boulder Clay* vs. *Tarn Flat*. Results on salinity range for growth of isolates were transformed and a cluster analysis was performed in order to compute a non-metric multi-dimensional scaling analysis (nMDS) by imposing as factor the sampling site (Figure 4c). The figure clearly shows the separate clustering of Actinobacteria and Bacteroidetes members from BC brine samples, which showed a more stenohaline behaviour.

### 3.3. Antibiotic Susceptibility and Heavy Metal Tolerance

#### 3.3.1. Antibiotic Susceptibility 

Overall, the susceptibility to antibiotics of bacterial isolates was in a different order among BC and TF brine samples. Isolates from BC brines showed a high susceptibility toward kanamicin and a good tolerance toward ampicillin and streptomycin. In general, TF4 brine strains were more tolerant to antibiotics than isolates from TF5, with ampicillin as the best tolerated antibiotic, while a great susceptibility was evidenced toward kanamicin and gentamycin. Multi-tolerance was observed for isolates from both BC and TF brine samples.

**Figure 5:**
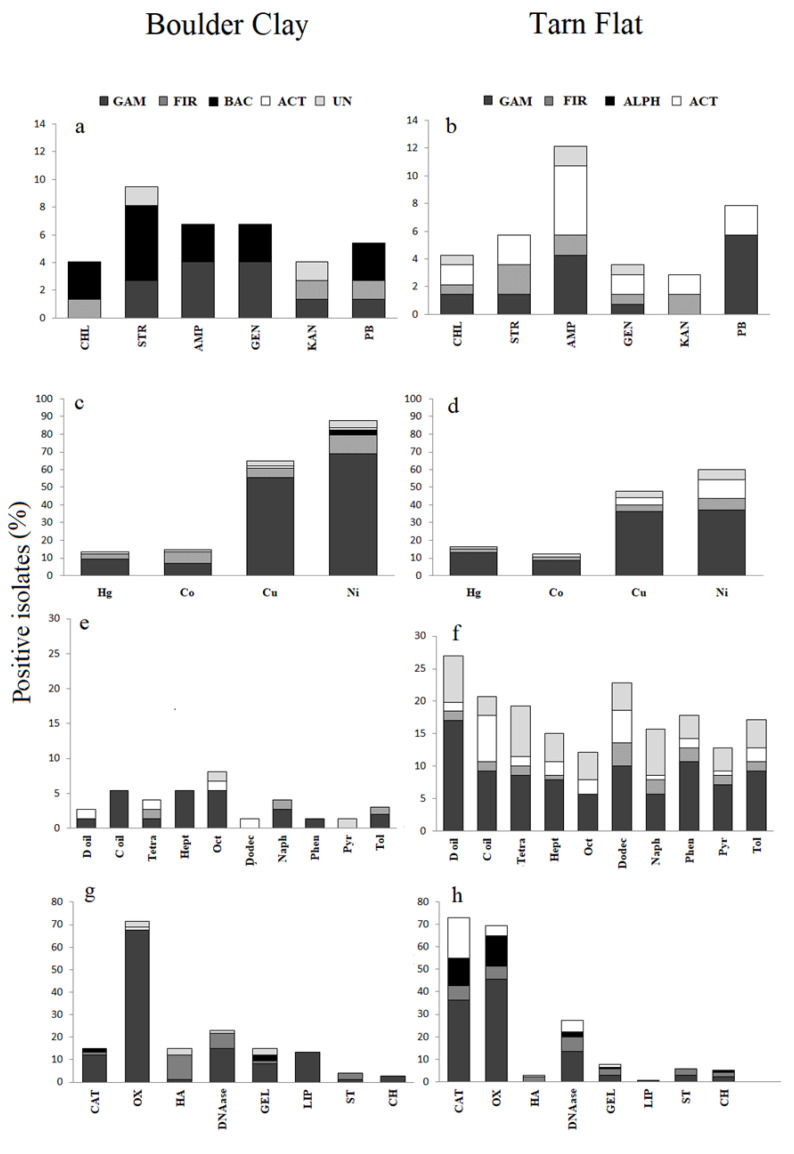
Ecological and biotechnological features of bacterial isolates. (**a**,**b**) Antibiotic susceptibility of BC and TF isolates (CHL, chloramphenicol; STR, streptomycin; AMP, ampicillin; GEN, gentamycin; KAN, kanamycin; PB, polymyxin B). (**c**,**d**) Resistance to heavy metals of BC and TF isolates (Hg, Mercury; Co, Cobalt; Cu, Copper; Ni, Nichel. (**e**,**f**) Oxidation of aliphatic and aromatic hydrocarbons by BC and TF isolates (D oil, Diesel oil; C oil, Crude oil; Tetra, Tetradecane; Hept, Heptane; Oct, Octane; Dodec, Dodecane; Naph, Naphtalene; Phen, Phenantrene; Pyr, Pyrene; Tol, Toluene. (**g**,**h**) Enzymes detected in BC and TF isolates (CAT, catalase; OX, oxidase; HA, haemolytic activity; DNAase, DNAse; GEL, gelatinase; LIP, lipase/esterase, ST, amylase; CH, chitinase.

*Boulder Clay*. The overall susceptibility to antibiotics of bacterial isolates from the three BC brine samples was in the order KAN > CHL > PB > GEN > AMP = STR, with Gammaproteobacteria and Bacteroidetes members that exhibited a higher antibiotic resistance (Figure 5a). Multi-resistance was often observed. 

For example, two *Gelidibacter* strains (namely, *Gelidibacter* spp. BC1-118A and BC1-118B) from BC1 brine resulted multi-resistant, being able to tolerate ampicillin, chloramphenicol, polymyxin and gentamycin (up to 50, 100, 25 and 350 ppm, respectively). *Staphylococcus* sp. BC1bis-71 was able to tolerate three antibiotics (chloramphenicol up to 100 ppm and both kanamycin and polymyxin B up to 25 ppm). All other isolates from BC1 tolerated one or two tested antibiotics. The few strains from BC3 brine samples resulted able to tolerate only one or two antibiotics and were generally affiliated to *Pseudomonas* spp. or *Psychrobacter* spp. 

*Tarn Flat*. The overall susceptibility to antibiotics of bacterial isolates from the two TF brines was in the order KAN > GEN > CHL > STR > PB > AMP, with Gammaproteobacteria and Actinobacteria members that exhibited a wider antibiotics resistance (Figure 5b). Four strains from TF4 (i.e., *Sporosarcina* sp. TF4-9, *Psychrobacter* sp. TF4-164, *Carnobacterium* sp. TF4-163 and *Leifsonia* sp. TF4-181) were able to tolerate streptomycin up to 25 ppm. *Sporosarcina* sp. TF4-68 and *Leifsonia* sp. TF4-14 were able to tolerate streptomycin and kanamycin, respectively, up to 350 ppm. Four strains (i.e., *Psychrobacter* sp. TF4-69, *Planococcus* sp. TF4-125, *Leifsonia* sp. TF4-181 and *Rhodobacter* sp. TF4-242) were resistant to ampicillin up to 100 ppm, while only *Marinobacter* sp. TF4-233 tolerated chloramphenicol up to 100 ppm. Multi-resistance was particularly evident for two *Leifsonia* isolates from TF5 brine (namely, TF5-105A and TF5-105B) that tolerated five and four antibiotics, respectively. Only three strains from TF5 brines (namely, *Leifsonia* spp. TF5-105A and TF5-105B and *Rhodobacter* sp. TF5-131) were resistant to chloramphenicol, while no strain tolerated gentamycin. 

#### 3.3.2. Heavy Metal Tolerance

Overall, HM resistance of bacterial isolates from analysed brines (BC plus TF) was in the order Ni > Cu > Hg/Co > Cd. Tolerance to Cd was never observed, while tolerance up to higher HM concentrations was observed mainly for isolates from TF4 and BC3 brine samples.

*Boulder Clay*. The relative HM-resistance in bacterial isolates from BC brines was in the order of Ni > Cu > Co > Hg > Cd (tolerance to Cd was never observed). Gammaproteobacteria members (mainly *Pseudomonas* isolates) generally appeared to be more tolerant than other isolates (Figure 5c). Overall, resistance to HMs, with a number of multi-tolerance cases, were evidenced evenly in BC1 and BC3 brine samples, proportionally to the number of isolates obtained from each of them. Among Hg-resistant strains from BC1, isolates in the genera *Carnobacterium*, *Psychrobacter* and *Staphylococcus* tolerated up to 1000 ppm of such metal. The remaining Hg-resistant strains were from BC3 brine and affiliated to the genera *Psychrobacter* (strains BC3-8, BC3-13, BC3-32, BC3-33 and BC3-97) and *Pseudomonas* (strain BC3-46). Four *Carnobacterium* strains from BC1 and five *Psychrobacter* spp. from BC3 were resistant to Co up to 500 ppm. Cu-resistant isolates mainly occurred the BC1 brine, tolerating up to 500 ppm of the metal and were related to *Pseudomonas* sp. (32 isolates), *Carnobacterium* sp. (4 isolates), *Shewanella* sp. (3 isolates), *Leifsonia* (1 isolate) and *Staphylococcus* sp. (1 isolate). The five Cu-resistant isolates from BC3, affiliated to *Psychrobacter*, grew up to 1000 ppm. Ni-resistant strains from BC1 resulted mainly affiliated to *Pseudomonas* spp., both up to 500 and 1000 ppm, while *Staphylococcus* BC1 bis-71 and *Gelidibacter* BC1-118B were able to tolerate Ni up to 2500 ppm. Three *Psychrobacter* strains from BC3 were able to tolerate Ni up to 1000 ppm, while other two were resistant to Ni up to 2500 ppm. Multi-resistance up to two metals was exhibited mainly by *Pseudomonas* spp. isolates (33 strains), *Shewanella* spp. (3 strains), *Carnobacterium* (4 strains) and one *Leifsonia* sp. isolate, from BC1 and BC3 brine samples. *Staphylococcus* sp. BC1bis-71 from BC1 and five *Psychrobacter* isolates from BC3 exhibited multi-resistance up to four different metals.

*Tarn Flat*. The relative resistance to the metals decreased in the order of Ni > Cu > Hg > Co > Cd (tolerance to Cd was never observed). HM resistance was especially observed in gammaproteobacterial isolates (Figure 5d), mainly in *Psychrobacter* affiliates that were able to tolerate almost all metals. Among Hg-resistant strains, *Psychrobacter* spp. isolates (eight strains), *Rhodobacter* spp. (two strains), *Sporosarcina* sp. and *Marinobacter* sp. were able to tolerate Hg up to 500 ppm, while 11 strains affiliated to *Pseudomonas* (five strains) and *Psychrobacter* (four strains), *Sporosarcina* and *Planococcus* (one strain each) tolerated Hg up to 1000 ppm. Such findings were mainly related to strains isolated from TF4, as only the isolate *Rhodobacter* sp. TF5-149 from TF5 showed resistance to Hg up to 500 ppm. Co and Cu were tolerated up to 500 ppm by respectively 17 and 38 strains, mainly affiliated to *Psychrobacter* and *Pseudomonas* genera. *Psychrobacter* spp. (21 strains), *Pseudomonas* spp. (five strains), *Leifsonia* sp., *Planococcus* sp., *Sporosarcina* sp. (one strain each) tolerated Cu up to 1000 ppm. Ni was the most tolerated metal, up to 5000 ppm (*Leifsonia* sp. TF4-181). A number of 40 strains from TF5 brines, mainly *Pseudomonas* spp., tolerated Ni up to 500 ppm and three and one strains from the same samples tolerated the metal up to 1000 ppm and 2500 ppm. Multi-resistance was observed for *Psychrobacter* and *Pseudomonas* spp. isolates able to tolerate two, three (isolates from both brine samples) and four (only isolated from TF4) different metals.

### 3.4. Biotechnological Potential of Bacterial Isolates

#### 3.4.1. Oxidation of Hydrocarbons

Overall, the growth in the presence of aliphatic hydrocarbons was observed for isolates from both BC and TF brines, with a higher extent for short chain hydrocarbons, such as *n*-tetradecane. Growth in the presence of aromatic compounds was mainly observed for isolates from TF brines.

*Boulder Clay*. Few strains grew in the presence of naphthalene and toluene as the sole carbon source (Figure 5e). Among BC1 isolates, only *Staphylococcus* strain BC1 bis-71 was able to use diesel oil. Four *Psychrobacter* isolates from BC3 (namely, BC3-8, BC-13, BC-32, BC-33) were able to grow in the presence of crude oil, heptane and octane, while *Psychrobacter* strain BC3-97 was able to use only diesel oil, toluene and phenanthrene. Among isolates growing on agar plates in the presence of hydrocarbon mixtures (6 isolates), only *Psychrobacter* spp. BC3-8 and BC3-97 were able to utilize crude oil and diesel oil in liquid cultures, respectively. 

*Tarn Flat*. TF brines (mainly Gammaproteobacteria) furnished a higher percentage of isolates able to grow in presence of hydrocarbons than BC ones. Diesel oil and dodecane were the most utilized carbon sources (26.9% and 21.3% of total strains, respectively) (Figure 5f). Overall, both TF4 and TF5 brines provided a high number of strains able to use aliphatic hydrocarbons (23 and 31 strains, respectively), followed by strains able to use oil mixtures (28 and 17 strains, respectively) and, finally, by strains able to oxidize aromatic hydrocarbons (30 and 22 for TF4 and TF5 brines, respectively). Naphthalene was mainly utilized by TF5 isolates affiliated to the genus *Rhodobacter* (13 strains) rather than by TF4 isolates (9 strains). Otherwise, TF4 provided a higher number of isolates able to grow in presence of diesel oil, with a total of 25 isolates mainly affiliated to the genus *Psychrobacter*. Among isolates growing on diesel oil- and crude oil-amended agar plates (37 and 17 isolates, respectively), five *Psychrobacter* (namely, TF4-31C, TF4-69, TF4-119, TF4-164, TF4-166) and *Marinobacter* sp. TF4-233 from TF4 brine grew also in liquid cultures, with most strains that were able to use diesel oil.

#### 3.4.2. Oxidation of Polychlorobiphenyls

Overall, the growth in presence of Aroclor 1242 was observed for 64 out of 215 isolates (25 and 39 from BC and TF brines, respectively; 29.8% of total isolates). 

*Boulder Clay*. PCB oxidation was observed for sixteen *Pseudomonas* strains and six *Psychrobacter* strains, in addition to *Shewanella* sp. BC1-22B, *Carnobacterium* sp. BC1-126 and *Staphylococcus* sp. BC1bis-71, even if they did not harbour the *bph*A gene.

*Tarn Flat*. PCB-oxidizing isolates from TF (all from TF5 brines) mainly belonged to the genera *Psychrobacter*, *Rhodobacter* and *Leifsonia* sp. (14, 7 and 7 strains, respectively), in addition to *Pseudomonas* and *Sporosarcina* isolates (5 and 4 strains, respectively). *Kocuria* sp. TF4-15 and *Marinobacter* sp. TF5-114C also resulted able to oxidize PCBs. Among them, the amplification of the *bph*A gene fragment gave a positive result for five *Rhodobacter* stains (namely, TF5-131, TF5-135, TF5-137, TF5-148 and TF5-224; *data not shown*).

#### 3.4.3. Extracellular Enzymes and Hydrolysis of Complex Substrata

Results for enzyme production and macromolecule hydrolysis are shown in Figure 5g–h. 

*Boulder Clay*. The 22.9% and 13.5% of total BC isolates, mainly isolates in the genera *Pseudomonas* and *Psychrobacter*, resulted able to hydrolyse DNA and tween 80, thus indicating the production of DNase and esterase/lipase, respectively. The presence of gelatinase occurred in the 16.2% of BC isolates, phylogenetically distributed among Gammaproteobacteria, Firmicutes and Bacteroidetes. Three strains (4.1% of total BC isolates) related to *Psychrobacter* sp. (strain BC1-70) and *Carnobacterium* sp. (strains BC1-73 and BC1-95) were amylase positive, while two strains (2.7%) affiliated to *Pseudomonas* sp. (strain BC1-1B) and *Shewanella* sp. (strain BC1-24) were chitinase positive. Agarolytic activity was never observed. 

*Tarn Flat*. A total of 38 strains (26.9% of total isolates; 23 and 15 isolates from TF4 and TF5, respectively) hydrolysed DNA. No strain showed agarolytic activity, whereas only *Psychrobacter* sp. TF4-146 possessed lipolytic activity on tween 80. The presence of gelatinase occurred in 11 strains (7.8% of total TF isolates). Among them, four were from TF4 and belonged to the genera *Sporosarcina* (strains TF4-9 and TF4-78), *Aeromicrobium* and *Planococcus* (one strain each, TF4-24 and TF4-125, respectively). Other seven strains were from TF5 and were related to the genera *Pseudomonas* (strains TF5-42B, TF5-44B, TF5-45A, TF5-45B), *Sporosarcina* (strain TF5-76), *Cryobacterium* (strain TF5-151) and *Rhodobacter* (strain TF5-219). Amylase occurred in eight strains (5.7% of total isolates) of which six were from TF4, mainly affiliated to the genera *Pseudomonas* (strains TF4-94, TF4-95, TF4-99 and TF4-100) and *Sporosarcina* (TF4-9 and TF4-68) and two from TF5 both affiliated to the genus *Sporosarcina* (strains TF5-66 and TF5-162). Chitinase activity was detected in seven strains mainly affiliated to the genera *Pseudomonas* (strains TF5-186, TF5-192B, TF5-196) and *Leifsonia* (strains TF4-31A, TF5-187, TF5-188).

#### 3.4.4. Other Enzymes

*Boulder Clay*. Haemolytic activity was exhibited by 11 isolates (14.8% of total isolates), mainly Firmicutes members. The haemolytic activity was evidenced by a dark and greenwash halo around the colonies, indicator of a non-complete haemolysis (alpha-haemolysis), performed by bacterial hydrogen peroxide which acted by oxidizing haemoglobin to green methaemoglobin (Figure 5g). Most BC isolates showed oxidase activity (71.6% of positive isolates on the total of BC isolates), with *Pseudomonas* and *Shewanella* members detected in BC1 brines, while *Psychrobacter* members from BC3 samples (Figure 5g). All strains from BC samples were mainly catalase negative (61 out of 74; 82.4%), with the exception of all *Psychrobacter* isolates (from BC1 and BC3), four *Pseudomonas*, one *Staphylococcus* and *Gelidibacter* isolate from BC1.

*Tarn Flat*. Haemolytic activity was exhibited only by four isolates (2.9% of total isolates) within Firmicutes and Actinobacteria, from TF4 brine, namely *Kocuria* sp. TF4-15 (which showed a beta haemolysis, sometimes called complete haemolysis, as it is a complete lysis of red cells in the medium) and *Carnobacterium* spp. (TF4-156, TF4-157 and TF4-163) (alpha haemolysis, a non-complete haemolysis, visible as dark and greenish halo around the colonies caused by hydrogen peroxide produced by the bacterium, that oxidizes haemoglobin to green methaemoglobin) (Figure 5h). The majority of isolates, mainly within Gammaproteobacteria, possessed catalase (72.3% of positive isolates on the total of TF brines isolates; 50 and 52 isolates from TF4 and TF5, respectively) and oxidase activities (68.8% of positive strains on the total of TF isolates; 48 and 49 isolates from TF4 and TF5, respectively) (Figure 5h).

#### 3.4.5. Inhibition of Bacterial Growth

None of the strains isolated from Antarctic lake brines showed inhibitory activity against target bacteria used in this study. 

#### 3.4.6. Production of Extracellular Polymeric Substances

Overall, a total of 42 isolates (19.5% of total isolates) showed a mucoid aspect on agar plates amended with glucose. Among them, the slime test was positive for four isolates, as follows.

*Boulder Clay*. Isolates *Pseudomonas* spp. BC1-139 and BC1bis-18 from BC1 brine produced EPS amounts of 170.1 and 80.2 µg EPS mL^−1^, respectively. 

*Tarn Flat*. EPS-producing isolates from BC brine belonged to the genera *Psychrobacter* (strain TF4-72) and *Pseudomonas* (strain TF5-192A). They produced EPS amounts of 68.7 and 20.5 µg EPS mL^−1^, respectively. 

### 3.5. Statistical Analyses

**Figure 6:**
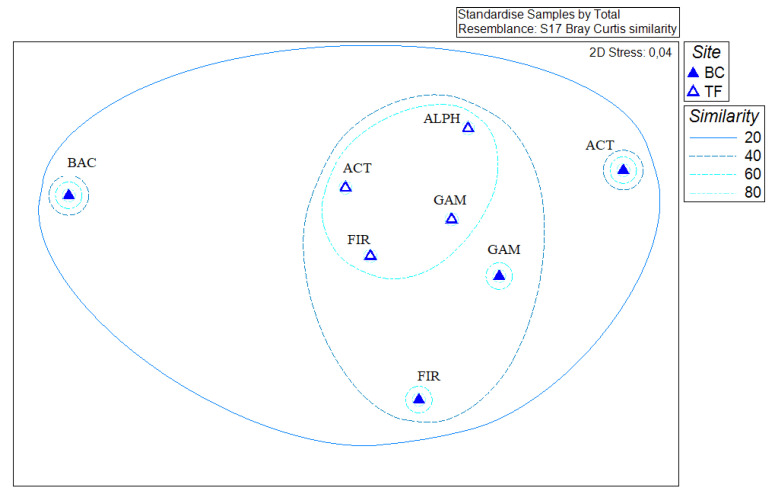
Non-metrical multidimensional scaling (nMDS) analysis (Bray-Curtis similarity matrix) computed on the entire phenotypic data set, evidencing connections with the phylogenetic affiliation of isolates.

Data on growth conditions, antibiotic susceptibility, HM-tolerance and biotechnological potentialities of bacteria isolated from Antarctic lake brines were statistically analysed in order to highlight any connection among analysed features, phylogenetic affiliation of isolates and brine samples (Figure 6). Actinobacteria and Bacteroidetes members from BC brine samples clustered separately, while a single cluster was constituted by all phylogenetic groups from TF brines, with a similarity of 80%. As it was evidenced, Gammaproteobacteria and Firmicutes from BC lakes had a global different pattern and resulted separated from the TF cluster but more similar one to each other than to Actinobacteria and Bacteroidetes members from BC brines. 

**Figure 7:**
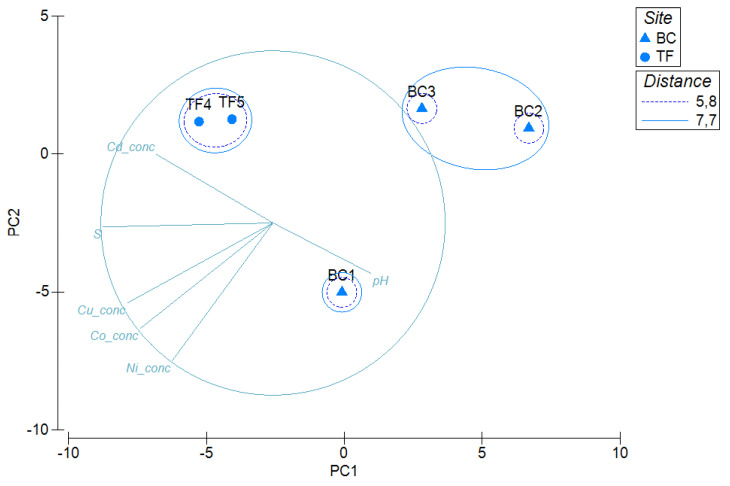
Principal component analysis computed on the entire phenotypic data set and environmental parameters, evidencing differences between brine samples.

A principal component analysis was computed on transformed data from the entire data set (including results from antibiotic susceptibility, HM-tolerance, oxidation of hydrocarbons and extracellular enzymes, hydrolysis of complex substrata and biotechnological potentialities) and environmental parameters (trace element concentrations, pH and salinity), by setting the site (BC and TF) as a factor. As it shown in Figure 7, TF brines clustered together and separately from BC brines. Among these latter, BC2 and BC3 brine samples formed a single group, which was separated from BC1. 

## 4. Discussion

Despite major advances have been made in the last decades, our current knowledge of physiology, metabolism, microbial ecology and interactions in cold-adapted microorganisms remains quite limited and it is particularly scarce with regard to less investigated habitats, such as Antarctic lake brines [56]. Brines of perennially ice-covered Antarctic lakes represent ideal environmental models to gain important insights on the ecology of the microbial life in Antarctica, by an overall synergistic analysis of both taxonomic diversity and functional capacities and interactions within a pristine ecosystem [13,57]. Recent culture-independent studies have shown that Antarctic brines in Boulder Clay and Tarn Flat lakes harbour diverse prokaryotic taxa, also highlighting astrobiological implications due to the strong similarities (e.g., harsh values of temperature and salinity) between these cryogenic habitats on Earth and other planetary and celestial bodies [13,31].

Differently, the current study was focused on the cultivable fraction of the cold-adapted bacterial community inhabiting Antarctic lake brines. Bacterial isolates were tested to first explore their response to contaminants, as well as their biotechnological potential as a source of novel biomolecules with applications, for example, for human health and environmental protection. Taking into consideration the different nature of the analysed brine samples, including salt concentration, different isolation media were used, with the richest medium in its most diluted composition (i.e., TSA) that resulted the most valuable one to obtain a larger data-set on both abundance and diversity of isolates. Overall, the CFU counts were comparable with those reported for Greenland and High Arctic glacier ice [58,59] and glacier cryoconite holes [60]. In line with our findings, several authors reported on the isolation of bacterial strains from frozen environments by using low nutrient or diluted media and long incubation periods at relatively low temperatures [59,60,61,62,63].

Similarly to previous culture-independent results [13,31], even if (as it was expected) at different relative percentages and distribution, the cultivable bacterial community was composed by Proteobacteria (namely Gamma- and Alphaproteobacteria), Bacteroidetes (only in BC brines), Actinobacteria (mainly in TF) and Firmicutes, with most phylotypes that are well known for inhabiting cold and salty environments [64,65,66]. Most isolates from TSA_100_ and TSA_50_ plates were affiliated to Gammaproteobacteria and Actinobacteria. These latter are of major importance in terrestrial and freshwater systems, better utilizing a number of carbon than other phylogenetic groups. Of note was the isolation of a high number of *Leifsonia* members from BC brines, strengthening that, as summarized by Baker et al. [67], psychrotolerant or psychrophilic *Leifsonia* species frequently originate from Antarctica. The predominance of Gammaproteobacteria has been frequently reported in polar systems [68,69,70,71,72] and in sub-glacial environments [73]. As r-strategists, Gammaproteobacteria generally occur at high frequency in culture collection due to their ability to rapidly grow on nutrient-rich media and successfully compete under heterotrophic conditions. The presence of *Psychrobacter* and *Marinobacter* members (which were among most halotolerant isolates, especially in TF brines) was previously reported in other cryogenic environments, such as sea-ice and icy coastal seawater [68,69,71,74,75], super cooled water brine lenses in permafrost derived from ancient marine layers of the Arctic Ocean [76,77] and accreted ice at the base of deep ice cores above seawater [68]. More interestingly, *Psychrobacter* and *Marinobacter* members, mainly occurring in TF4 samples, also isolated from brines of the Antarctic Lake Vida [1,78,79]. Among Proteobacteria, the alphaproteobacterial genera *Devosia* and *Rhodobacter* were well represented in TF brines. The former was previously detected in alpine glacier cryoconite holes [80], while *Rhodobacter* halophilic affiliates were reported in relation to hydrocarbon oxidation in saline systems (see below). Bacteroidetes, which were highly abundant by the culture-independent approach [13,31], occurred at low percentages within the analysed cultivable fraction of BC brines (and were absent in TF brines), suggesting that the culture media used were not suitable for their growth under laboratory conditions. Finally, among Firmicutes the genera *Carnobacterium* and *Sporosarcina* were previously retrieved in other Antarctic environments, such as Antarctic lakes and ponds [79,81,82,83] and Arctic permafrost ice [84].

In this study, chemical analyses focussed on heavy metals and organic pollutants revealed that lake brines were almost unaffected by anthropogenic contamination. However, it is noteworthy that the construction of a semi-permanent gravel runway for airfreight operations is on-going in the Boulder Clay area at the time of writing but sampling was performed before any construction operations. Therefore, our data may represent the baseline for future environmental monitoring programmes aimed at evaluating, for preservation purposes, the potential shift in the response of autochthonous bacteria (in term of activities and diversity) in Antarctic lake brines following an unavoidable anthropogenic impact (hydrocarburic substances and heavy metals are expected to likely derive from airfreight construction and operations).At sampling time, contrary to organic pollutants whose concentration was <LOD, heavy metals were detected at low concentrations, suggesting that they could more likely derive from natural sources and processes, for example, rock leaching, sediments and snow meltwater. Both the bio-essentiality and abuse of metals, as well as antibiotics, can led to bacterial resistance phenomena. These latter are ubiquitous and have been also detected in bacteria from Antarctic seawater, marine sediment and benthic organisms, as well as freshwater environments and soils [85,86,87]. Tolerance towards heavy metals (resulting particularly evident in isolates from TF brines) was higher for nickel and copper than for mercury and cobalt, thus appearing strictly related to the determined metal concentrations. Similarly, the observed high susceptibility to cadmium of our Antarctic isolates was probably dependent on the absence of this metal in the analysed brine samples. Further reinforcing results from the chemical analyses, our bacterial isolates showed a high susceptibility to antibiotics, thus indirectly indicating the low or absent antibiotic load due to human or animal presence in both areas at sampling time. In fact, contrary to other Antarctic sites [88,89], our results led to exclude also the influence of airborne bacteria and migration birds as biological vectors of dissemination of antibiotic-resistant bacteria and genes from long distances to the analysed brine systems. All together, these findings suggest that bacteria might be used as bioindicators for tracking human footprints in these Antarctic areas. At this regard, further analyses are forecasted on heavy-metal tolerance and antibiotic susceptibility in bacteria isolated from BC brines during the airfreight construction.

Bacterial isolates were also screened for their ability to grow in presence of hydrocarbons and PCBs in order to explore the eventual utilization of autochthonous microorganisms (as the Antarctic Treaty forbids the introduction of non-native species) for bioremediative purpose in Antarctica. There are very few studies regarding organic contaminants in Antarctic lakes ([14] and references therein). Beside hydrocarbons of natural origin, petroleum hydrocarbon contamination, especially in localized areas (the Boulder Clay area is an example), is potentially the most likely source of anthropogenic pollution in Antarctic ecosystems [90]. According to Yao et al. [91] the composition profiles of polycyclic aromatic hydrocarbons in Antarctic inland lakes clearly derive from local human activities (e.g., local oil spills), rather than long-range transport, with data that are corroborated by several reports on recent fuel spillage from ship and plane crash incidents. Antarctic environments (with a lot of studies that have been carried out in seawater, marine sediment and soils) harbour indigenous hydrocarbon-oxidizing bacteria, whose abundances increase in hydrocarbon contaminated than in pristine sites and whose activity is lowered and slowed down in cold areas [42,92]. In this study, different patterns of hydrocarbon utilization at low temperature were observed (with aliphatic hydrocarbons, especially *n*-tetradecane, resulting more utilized than aromatics), suggesting that the different substrate specificities may lead to a more efficient degradation of complex hydrocarburic mixtures by a bacterial consortium in nature. Hydrocarbon-oxidizing isolates were mainly related to the genera *Psychrobacter*, *Rhodobacter* and *Marinobacter*, all frequently reported in relation to hydrocarbon-utilization (both aliphatic and aromatic hydrocarbons) also in Antarctica [42,93]. In particular, *Marinobacter* is among a few marine bacterial genera (i.e., *Alcanivorax*, *Thallasolituus*, *Cycloclasticus* and *Oleispira*) that include obligate hydrocarbon utilizer species [94,95]. A higher number of hydrocarbon-oxidizing bacteria was retrieved in TF (more saline) than in BC brines. Most of them were *Psychrobacter* and *Rhodobacter* affiliates able to growth up to 11–19% NaCl. According to Radwan and Al-Mailem [95], a halo-stress may enhance the biodegradative removal of hydrocarbon pollutants in saline and hyper-saline environments.

Contrary to hydrocarbons, PCBs are long-term persistent synthetic compounds. Since 1970s, their use and production have been regulated or prohibited in western nations. However, the occurrence of PCBs has been reported in a number of biotic and abiotic Antarctic matrices (e.g., soils, benthic organisms, sediment and seawater) [96,97,98,99]. Recently, Vecchiato et al. [14] suggested that the lakes of Northern Victoria Land are not yet heavily affected by POP (including PCBs) contamination. On the other hand, data on PCB-degrading Antarctic cold-adapted bacteria are quite scarce and limited to few studies reporting their occurrence in seawater and sediment at Terra Nova Bay [38,98,99]. The isolation of PCB-oxidizing bacteria from Antarctic lake brines is here reported for the first time. The percentage of bacteria from brines that were able to grow in presence of the PCB mixture Aroclor 1242 was consistently lower than that previously observed for marine sediment (21.4%) [38] but it was comparable, even if slightly lower, to that reported for bacteria from seawater (71%) [46]. The presence of the *bph*A gene portion, encoding for the first fundamental step in the biphenyl upper pathway and confirming the ability to aerobically degrade PCBs, was detected only in four PCB-oxidizing *Rhodobacter* isolates from TF brines. However, high biodegradation potential is often shown also by isolates that did not seem to harbour the gene [47].

The utilization of hydrocarbons and PCBs is often enhanced by the production of biosurfactants [92]. However, in this study isolates showing haemolytic activity, as a useful indication of the possible synthesis of anionic surfactants, were not among those able to growth in presence of such contaminants. Both in the case of hydrocarbon- and PCB-oxidizing isolates, future analyses will be addressed to the relation between the occurrence of the catabolic genes and the biodegradation efficacy at in situ temperatures. Promising results were indeed obtained from biodegradation experiments with two brine isolates (i.e., *Psychrobacter* sp. BC3-97 and *Staphylococcus* sp. BC1 bis-71) on Aroclor 1242, with degradation efficiency of more than 80% after incubation at 15 °C (*data not shown*).

The search for new resources in remote and particularly unexplored environments is increasingly becoming the main key to enhancing biotechnological potential of natural basin and expanding prospects in emerging areas. Beside the study on the response of Antarctic bacterial isolates to heavy metals and antibiotics, as well as the potential use of autochthonous bacteria for the attenuation of the contamination by hydrocarbons and polychlorobiphenyls in cold systems, here we report on the first attempt to bioprospect bacterial isolates from Antarctic lake brines for cold-active enzymes, antibiotics and extracellular polymeric substances to be applied in biotechnological fields. Differently from results obtained for bacteria isolated from other Antarctic matrices (i.e., seawater, marine sponges, lake sediments, microbial mats, permafrost and soils) [50,51,100,101,102,103,104], our strains showed any inhibitory activity against the bacterial pathogens used as a target in this study. This finding might be dependent on a number of causes, such as the absence of antagonistic stimuli in their natural environment or the thermolability of the eventually produced antimicrobial compounds.

Cold-active enzymes have potential applications in a broad range of biotechnological processes (including industrial, agricultural and pharmaceutical ones) [105]. The bacterial community in the analysed Antarctic brines may represent a novel source of biotechnologically exploitable cold-active enzymes. Most isolates produced one or more of the enzymes among amylase, lipase/esterase, gelatinase, chitinase and DNase at low temperature. DNase is a powerful research tool for DNA manipulations and the treatment of bacterial biofilm infections. DNase positives were more frequent than other enzymes in bacterial isolates from both BC and TF brines and they were primarily distributed among *Pseudomonas* isolates. The degradation of extracellular DNA provides C, N and P sources for prokaryotic metabolisms and DNA may represent a crucial trophic resource in briny ecosystems. Bacterial producing lipases and proteases (we screened bacteria for gelatinase) may play key roles in the mineralization of the organic matter. In fact, the occurrence of bacteria able to produce gelatinase suggested that organic nitrogen may be degraded in brines, while lipolysis, together with chitinolysis, in the brine samples may indicate the degradation of zooplankton components, for example, lipids (the most significant zooplankton fraction) and chitin (in the zooplankton exoskeletons). Finally, polysaccharide hydrolysis indicated that bacteria may be involved in the decomposition of exopolymeric substance and phytoplankton detritus.

Surprisingly, taking into consideration the cryo-features of brines, the number of isolates able to produce EPS (also involved in the cryoprotection process) was quite low. However, most promising isolates, mainly belonging to the well-known biofilm-producing genus *Pseudomonas*, merit to be analysed more in depth. In fact, to date very few Antarctic EPS-producing bacterial species have been characterized and little is known about the chemical structures of the extracellular polymeric substances produced [106].

## 5. Conclusions

Although the cultivation methods have the disadvantage of losing a substantial fraction of a bacterial community, they provide the opportunity to perform deeper analysis on preserved strains, gaining information that could not be gained directly from sequencing efforts alone. Results obtained from this study on the physiological and enzymatic potential of the cold-adapted isolates from Antarctic lake brines provide interesting prospects for possible applications in the biotechnological field through future targeted surveys, for example, the high throughput screening for bioactive metabolites (e.g., enzymes, EPSs, biosurfactants) and their purification and characterization, as well as the exploration of biodegradation efficiency at low temperatures or the response of bacterial communities to the increasing presence of anthropogenic contaminants.

## Figures and Tables

**Table 1:** Boulder Clay and Tarn Flat viable counts (CFU mL^−1^ × 10^3^) and number of isolates (in brackets) obtained from direct plating per sample and isolation medium.

	Viable Counts (Number of Isolates) on Different Culture Media ^1^					
	TSA_1_	TSA_50_	TSA_100_	DSMZ97	R2A_10_	Isolates (*n.*)
Boulder Clay						
BC1	15.5 ± 0.7 (38)	4.3 ± 0.9 (15)	5.3 ± 0.8 (11)	0.0 ± 0.0	0.0 ± 0.0	64
BC2	0.0 ± 0.0	0.5 ± 0.3 (1)	0.1 ± 0.8 (1)	0.0 ± 0.0	0.0 ± 0.0	2
BC3	0.0 ± 0.0 (2)	0.9 ± 0.3 (5)	2.3 ± 0.8 (1)	0.1 ± 0.7 (0)	0.0 ± 0.0	8
Tarn Flat						
TF4	3.1 ± 0.8 (14)	4.7 ± 0.9 (16)	3.3 ± 0.1 (31)	0.0 ± 0.0	0.1 ± 0.0 (5)	66
TF5	7.2 ± 0.6 (41)	5.5 ± 0.0 (14)	5.5 ± 0.8 (16)	0.0 ± 0.0	0.1 ± 0.0 (4)	75
Isolates (*n.*)	93	51	62	0	9	215

^1^ TSA_100_, TSA_50_ and TSA_1_—plates of TSA at full, 50% and 1% strength respectively.

**Table 2:** 16S rRNA gene sequence affiliation to their closet phylogenetic neighbours of bacterial isolates from Antarctic lake brines. Numbers in brackets indicate the number of isolates per medium. Unidentified isolates are not listed.

Next Relative by Gen Bank Alignment (AN ^a^, Organisms)	RI ^b^	AN ^a^	OUT ^c^	Hom (%) ^d^	Isolates (*n*.)			Isolation Medium ^e^		
					BC1	BC2	BC3	BC1	BC2	BC3
Boulder Clay										
Gammaproteobacteria										
JQ229609, *Pseudomonas* sp. ALI-cc18	BC1-5	MT350301	24	99	42		1	B (10), C (32)		A
DQ677864, *Psychrobacter* sp. BF01 S5	BC3-33	MT350302	27	99	1		5	B		B
KU179857, *Shewanella arctica* RKAT059	BC1-22B	MT350303	28	99	3			A		
CF group of Bacteroidetes										
HQ538744, *Gelidibacter gilvus* strain Z20	BC1-72A	MT350304	na	96	1			A		
AF001367, *Gelidibacter algens*	BC1-118A	MT350305	30	98	2			A		
FR772078, *Flavobacterium* sp. R-40832	BC2-39	MT350306	31	99		2			A (1), B (1)	
Actinobacteria										
KT965169, *Leifsonia* sp. N10	BC1bis-11	MT350307	na	98	1			C		
Firmicutes										
LC145583, *Carnobacterium* JCM 12498	BC1-54	MT350308	22	99	9			A (6), B (3)		
LT221233, *Staphylococcus* LK1HaP1	BC1bis-71	MT350309	na	99	1			A		
**Tarn Flat**	**RI ^b^**	**AN ^a^**	**OTU ^c^**	**Hom (%) ^d^**	**TF4**	**TF5**		**TF4**	**TF5**	
Alphaproteobacteria										
KR140230, *Devosia psychrophila* strain NJES-30 16S	TF5-10A	KY437988	3	99		4			C	
AF513400, *Rhodobacter* sp. 1-5	TF5-149	KY437996	13	99	2	15		A, B	C (1), B (8), A (6)	
EU369117, Uncultured clone MBIOS-01	TF5-35A	KY438003	na	96		1			C	
Gammaproteobacteria										
KF384120, *Marinobacter* sp. LV10R510-5	TF4-237	KY437991	6	99	7	8		D (5), B (2)	D (4), B (1), A (3)	
KU749990, *Pseudomonas stutzeri* strain 1005	TF4-100	KY437993	8	99	4			B		
DQ677869, *Pseudomonas* sp. BF02_S14	TF4-182	KY437992	9	99	2	19		A, C	C (16), B (1), A (2)	
KX417186, *Psychrobacter fozii* strain 9.22	TF4-146	KY437994	10	100	33			A (22), B (7), C (4)		
DQ677864, *Psychrobacter* sp. BF02_S5	TF4-164	KY437995	na	96	1			A		
Actinobacteria										
FJ196003, *Aeromicrobium* sp. ZS1-19	TF4-24	KY437986	1	99	1	4		C	C	
KM507649, *Kocuria* sp. FXJ8.237	TF4-15	KY438002	na	99	1			C		
KC478079, *Leifsonia* sp. FO17	TF5-105B	KY437990	4	99	6	14		C	A (3), C (11)	
KF295312, *Rhodoglobus* sp. Y4_509_3	TF5-20A	KY437989	na	96		1			C	
AM183255, *Rothia* sp. BBH4	TF5-35B	KY438000	na	99		1			C	
DQ521554, uncultured bacterium ANTLV9_C09	TF5-151	KY437999	na	99		1			A	
**Next Relative by Gen Bank Alignment** **(AN ^a^, Organisms)**	**RI ^b^**	**AN ^a^**	**OUT ^c^**	**Hom (%) ^d^**	**Isolates (*n.*)**			**Isolation Medium ^e^**		
					**TF4**	**TF5**		**TF4**	**TF5**	
Firmicutes										
KF151857, *Alkalibacterium kapii* strain MGR70	TF4-239	KY438001	na	97	1			B		
LC145583, *Carnobacterium alterfunditum* JCM 12498	TF4-156	KY437987	2	99	3			A		
FR691465, *Planococcus antarcticus* strain R-36948	TF4-125	KY437998	na	99	1			A		
HM224487, *Sporosarcina* sp. TPD39	TF4-168	KY437997	15	99	4	7		A (2), B (1), C (1)	A (1), B (4), C (2)	

^a^ AN—accession number; ^b^ RI—representative isolate; ^c^ na—not assigned; ^d^ Hom: sequence homology; ^e^, A: TSA_100;_ B: TSA_50_, C: TSA_1_ (plates of TSA at full, 50% and 1% strength, respectively); D: R2A_10_ (plates of R2A at 10% strength).
